# *N*,*N*′-[(2*E*,3*E*)-Butane-2,3-diylidene]bis[4-fluoro-2-(1-phenyl­eth­yl)aniline]

**DOI:** 10.1107/S1600536814002657

**Published:** 2014-02-12

**Authors:** Juanjuan Wei, Houde She, Lijun Shi, Ziqiang Lei

**Affiliations:** a Key Laboratory of Eco-Environment-Related Polymer Materials of the Ministry of, Education, Key Laboratory of Polymer Materials of Gansu Province, College of Chemistry & Chemical Engineering, Northwest Normal University, Lanzhou 730070, People’s Republic of China

## Abstract

The title mol­ecule, C_32_H_30_F_2_N_2_, a product of the condensation reaction of butane-2,3-dione and 4-fluoro-2-(1-phenyl­eth­yl)aniline, is located about an inversion centre. In the asymmetric unit, the dihedral angle between the planes of the benzene and phenyl rings is 84.27 (5)°. Neither hydrogen bonding nor aromatic stacking is observed in the crystal structure.

## Related literature   

For the synthesis of α-di­imine ligands, see: Grasa *et al.* (2001 ▶); Williams *et al.* (2008 ▶); Hanhan *et al.* (2012 ▶); Partyka (2011 ▶); Yuan *et al.* (2012 ▶). For related structures, see: Zou *et al.* (2008 ▶); Lohr *et al.* (2011 ▶).
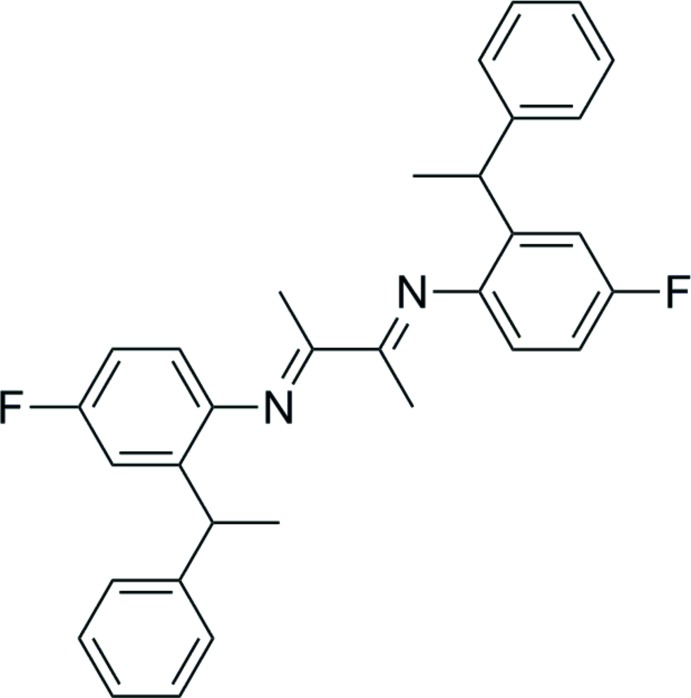


## Experimental   

### 

#### Crystal data  

C_32_H_30_F_2_N_2_
*M* *_r_* = 480.58
Monoclinic, 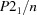

*a* = 11.5335 (11) Å
*b* = 9.5024 (12) Å
*c* = 12.1318 (14) Å
β = 91.660 (11)°
*V* = 1329.0 (3) Å^3^
*Z* = 2
Cu *K*α radiation
μ = 0.64 mm^−1^
*T* = 295 K
0.35 × 0.28 × 0.26 mm

#### Data collection  

Bruker APEXII CCD diffractometer
Absorption correction: multi-scan (*SADABS*; Bruker, 2008 ▶) *T*_min_ = 0.808, *T*_max_ = 0.852
5982 measured reflections
2507 independent reflections
2132 reflections with *I* > 2σ(*I*)
*R*_int_ = 0.019

#### Refinement  

*R*[*F* ^2^ > 2σ(*F* ^2^)] = 0.040
*wR*(*F* ^2^) = 0.118
*S* = 1.07
2507 reflections
166 parameters
H-atom parameters constrained
Δρ_max_ = 0.18 e Å^−3^
Δρ_min_ = −0.15 e Å^−3^

### 

Data collection: *APEX2* (Bruker, 2008 ▶); cell refinement: *SAINT* (Bruker, 2008 ▶); data reduction: *SAINT*; program(s) used to solve structure: *SHELXS97* (Sheldrick, 2008 ▶); program(s) used to refine structure: *SHELXL97* (Sheldrick, 2008 ▶); molecular graphics: *SHELXTL* (Sheldrick, 2008 ▶); software used to prepare material for publication: *SHELXTL*.

## Supplementary Material

Crystal structure: contains datablock(s) I, New_Global_Publ_Block. DOI: 10.1107/S1600536814002657/rk2419sup1.cif

Structure factors: contains datablock(s) I. DOI: 10.1107/S1600536814002657/rk2419Isup2.hkl

Supporting information file. DOI: 10.1107/S1600536814002657/rk2419Isup3.cml

CCDC reference:

Additional supporting information:  crystallographic information; 3D view; checkCIF report

## Figures and Tables

**Fig. 1.**
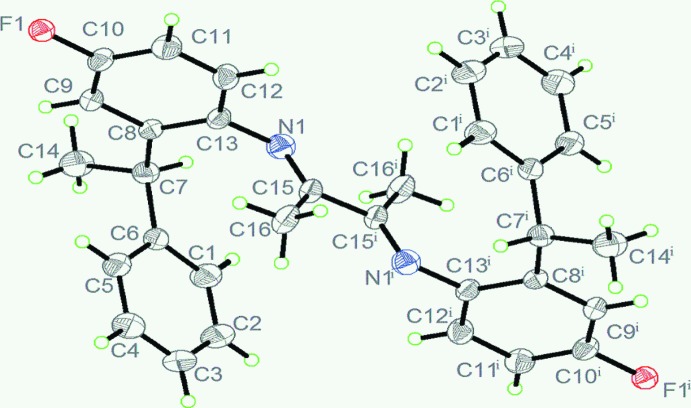
The molecular structure of title molecule with the atom numbering scheme. Displacement ellipsoids are drawn at 30% probability level. Symmetry code: (i) -x+1, -y+1, -z+1.

**Fig. 2.**
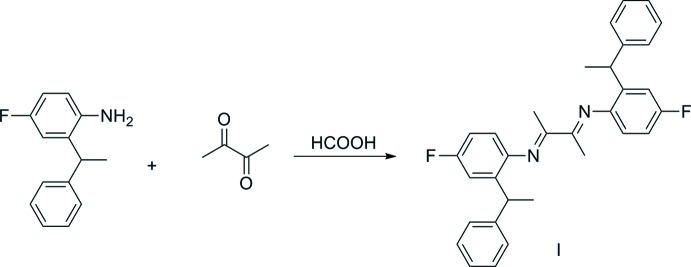
A condensation reaction of 2,3-butanedione and 4-fluoro-2-(1-phenylethyl)aniline.

## supplementary crystallographic information

### 1. Comment

An α-diimine ligands with different electronic property, rigidity and steric hindrance has been widely synthesized due to their significant applications in catalysis, coordination chemistry and carbene chemistry (Grasa *et al.*, 2001; Williams *et al.*, 2008; Hanhan *et al.*, 2012; Partyka, 2011; Yuan *et al.*, 2012). As part of our research efforts focused on developing ligands and organic catalysts, a novel series of imine derivatives were synthesized. Herein, we report the preparation and crystal structure of the title compound (Fig. 1).

The title molecule, is a symmetrical structure, and the central butanediimine moiety (N═C(*Me*)–C(*Me*)═N) is planar. The dihedral angles between the benzene ring C8-C13 and benzene ring C1-C6 is 84.27 (5)°. The molecular dimensions in the title compound agree very well with the corresponding one reported in a few closely related compounds (Zou *et al.*, 2008; Lohr *et al.* 2011).

### 2. Experimental

Formic acid (1.0 ml) was added to a stirred solution of 4-fluoro-2-(1-phenylethyl)aniline (1.5 mmol) and 2,3-butanedione (0.7 mmol) in 20 ml anhydrous methanol (20 ml). The mixture was stirred at 323 K for 24 h, then cooled, and the precipitate was separated by filtration. The solid was recrystallized from ethanol/dichloromethane (*v*/*v* = 12:1), washed with cold ethanol and dried under vacuum to give the title compound (Fig. 2). Yield is 82%. Crystals suitable for X-ray diffraction were grown in cyclohexane/dichloromethane (*v*/*v* = 1:2) solution at room temperature by the slow evaporation method. ^1^H NMR (400 MHz, CDCl_3_): δ (p.p.m.) 7.19(t, *J* = 7.4 Hz, 4H), 7.09-7.13 (m, 4H), 7.01-7.05(m, 4H), 6.93 (dt, *J* = 8.4, 2.9 Hz, 2H), 6.47 (dd, *J* = 8.6, 5.3 Hz, 2H), 4.08 (q, *J* = 6.8 Hz, 2H), 1.62 (s, 6H), 1.56 (d, *J* = 7.2 Hz, 6H). ^13^C NMR (100 MHz, CDCl_3_): δ (p.p.m.) 168.54, 161.22, 158.82, 145.58, 144.69, 137.34, 128.31, 127.57, 126.06, 118.94, 113.58, 40.17, 21.42, 20.86.

### 3. Refinement

All hydrogen atoms were placed in calculated positions with C–H distances of 0.93Å, 0.96Å and 0.98Å for aryl, methyl and methine H atoms. They were included in the refinement in a riding model approximation, respectively with *U*_iso_ = 1.2*U*_eq_(C) for aryl and methine H, and *U*_iso_ = 1.5*U*_eq_(C) for methyl H.

### Crystal data

**Table:**

C_32_H_30_F_2_N_2_	*F*(000) = 508
*M**_r_* = 480.58	*D*_x_ = 1.201 Mg m^−^^3^
Monoclinic, *P*2_1_/*n*	Cu *K*α radiation, λ = 1.54184 Å
*a* = 11.5335 (11) Å	Cell parameters from 2620 reflections
*b* = 9.5024 (12) Å	θ = 5.2–70.8°
*c* = 12.1318 (14) Å	µ = 0.64 mm^−^^1^
β = 91.660 (11)°	*T* = 295 K
*V* = 1329.0 (3) Å^3^	Block, clear light yellow
*Z* = 2	0.35 × 0.28 × 0.26 mm

### Data collection

**Table:**

Bruker APEXII CCD diffractometer	2507 independent reflections
Radiation source: fine-focus sealed tube	2132 reflections with *I* > 2σ(*I*)
Graphite monochromator	*R*_int_ = 0.019
φ and ω scans	θ_max_ = 70.5°, θ_min_ = 5.2°
Absorption correction: multi-scan (*SADABS*; Bruker, 2008)	*h* = −14→12
*T*_min_ = 0.808, *T*_max_ = 0.852	*k* = −11→10
5982 measured reflections	*l* = −14→14

### Refinement

**Table:**

Refinement on *F*^2^	Secondary atom site location: difference Fourier map
Least-squares matrix: full	Hydrogen site location: inferred from neighbouring sites
*R*[*F*^2^ > 2σ(*F*^2^)] = 0.040	H-atom parameters constrained
*wR*(*F*^2^) = 0.118	*w* = 1/[σ^2^(*F*_o_^2^) + (0.056*P*)^2^ + 0.1881*P*] where *P* = (*F*_o_^2^ + 2*F*_c_^2^)/3
*S* = 1.07	(Δ/σ)_max_ < 0.001
2507 reflections	Δρ_max_ = 0.18 e Å^−^^3^
166 parameters	Δρ_min_ = −0.15 e Å^−^^3^
0 restraints	Extinction correction: *SHELXL97* (Sheldrick, 2008), Fc^*^=kFc[1+0.001xFc^2^λ^3^/sin(2θ)]^-1/4^
Primary atom site location: structure-invariant direct methods	Extinction coefficient: 0.0100 (10)

### Special details

**Table:**

Geometry. All s.u.'s (except the s.u. in the dihedral angle between two l.s. planes) are estimated using the full covariance matrix. The cell s.u.'s are taken into account individually in the estimation of s.u.'s in distances, angles and torsion angles; correlations between s.u.'s in cell parameters are only used when they are defined by crystal symmetry. An approximate (isotropic) treatment of cell s.u.'s is used for estimating s.u.'s involving l.s. planes.
Refinement. Refinement of *F*^2^ against ALL reflections. The weighted *R*-factor *wR* and goodness of fit *S* are based on *F*^2^, conventional *R*-factors *R* are based on *F*, with *F* set to zero for negative *F*^2^. The threshold expression of *F*^2^ > σ(*F*^2^) is used only for calculating *R*-factors(gt) *etc*. and is not relevant to the choice of reflections for refinement. *R*-factors based on *F*^2^ are statistically about twice as large as those based on *F*, and *R*-factors based on ALL data will be even larger.

### Fractional atomic coordinates and isotropic or equivalent isotropic displacement parameters (Å^2^)

**Table:**

	*x*	*y*	*z*	*U*_iso_*/*U*_eq_	
N1	0.37261 (9)	0.49441 (12)	0.57384 (9)	0.0406 (3)	
F1	−0.02542 (7)	0.78857 (12)	0.66670 (9)	0.0687 (3)	
C1	0.57482 (13)	0.64883 (19)	0.75155 (16)	0.0602 (4)	
H1	0.5841	0.5539	0.7683	0.072*	
C2	0.67019 (14)	0.7270 (2)	0.72255 (19)	0.0747 (6)	
H2	0.7426	0.6844	0.7197	0.090*	
C3	0.65862 (15)	0.8671 (2)	0.69791 (18)	0.0743 (6)	
H3	0.7224	0.9194	0.6769	0.089*	
C4	0.55119 (15)	0.9295 (2)	0.70466 (16)	0.0687 (5)	
H4	0.5430	1.0251	0.6899	0.082*	
C5	0.45519 (13)	0.85116 (16)	0.73331 (14)	0.0543 (4)	
H5	0.3832	0.8946	0.7371	0.065*	
C6	0.46540 (11)	0.70923 (15)	0.75624 (11)	0.0420 (3)	
C7	0.36098 (11)	0.61988 (15)	0.78526 (11)	0.0419 (3)	
H7	0.3860	0.5215	0.7814	0.050*	
C8	0.26187 (10)	0.63660 (13)	0.70095 (10)	0.0361 (3)	
C9	0.16094 (11)	0.71082 (15)	0.72229 (11)	0.0422 (3)	
H9	0.1525	0.7555	0.7898	0.051*	
C10	0.07376 (11)	0.71744 (15)	0.64271 (13)	0.0468 (3)	
C11	0.08083 (12)	0.65451 (17)	0.54169 (13)	0.0518 (4)	
H11	0.0199	0.6601	0.4899	0.062*	
C12	0.18142 (12)	0.58231 (17)	0.51907 (11)	0.0488 (4)	
H12	0.1885	0.5385	0.4510	0.059*	
C13	0.27209 (11)	0.57440 (14)	0.59672 (10)	0.0379 (3)	
C14	0.32418 (14)	0.6453 (2)	0.90437 (13)	0.0628 (5)	
H14A	0.3068	0.7433	0.9142	0.094*	
H14B	0.3862	0.6185	0.9545	0.094*	
H14C	0.2566	0.5902	0.9188	0.094*	
C15	0.44839 (10)	0.54573 (14)	0.51063 (10)	0.0399 (3)	
C16	0.44521 (14)	0.68862 (16)	0.45826 (14)	0.0558 (4)	
H16A	0.3815	0.7414	0.4862	0.084*	
H16B	0.4356	0.6788	0.3798	0.084*	
H16C	0.5165	0.7371	0.4753	0.084*	

### Atomic displacement parameters (Å^2^)

**Table:**

	*U*^11^	*U*^22^	*U*^33^	*U*^12^	*U*^13^	*U*^23^
N1	0.0385 (6)	0.0450 (6)	0.0388 (6)	0.0027 (5)	0.0096 (4)	−0.0018 (5)
F1	0.0418 (5)	0.0843 (7)	0.0801 (7)	0.0223 (4)	0.0027 (4)	−0.0130 (5)
C1	0.0394 (7)	0.0582 (10)	0.0828 (11)	0.0038 (7)	−0.0031 (7)	−0.0062 (8)
C2	0.0369 (8)	0.0801 (13)	0.1072 (16)	−0.0020 (8)	0.0043 (9)	−0.0174 (11)
C3	0.0512 (9)	0.0828 (13)	0.0896 (13)	−0.0260 (9)	0.0173 (9)	−0.0201 (11)
C4	0.0681 (11)	0.0520 (10)	0.0868 (13)	−0.0131 (8)	0.0148 (10)	−0.0048 (8)
C5	0.0465 (8)	0.0472 (8)	0.0698 (10)	0.0001 (6)	0.0093 (7)	−0.0048 (7)
C6	0.0372 (6)	0.0461 (8)	0.0427 (7)	−0.0009 (5)	−0.0001 (5)	−0.0071 (6)
C7	0.0396 (7)	0.0438 (7)	0.0423 (7)	−0.0011 (5)	0.0004 (5)	0.0002 (6)
C8	0.0328 (6)	0.0381 (7)	0.0377 (6)	−0.0033 (5)	0.0066 (5)	−0.0001 (5)
C9	0.0387 (6)	0.0454 (7)	0.0431 (7)	0.0000 (5)	0.0085 (5)	−0.0065 (5)
C10	0.0341 (6)	0.0484 (8)	0.0583 (8)	0.0063 (5)	0.0066 (6)	−0.0022 (6)
C11	0.0407 (7)	0.0626 (9)	0.0515 (8)	0.0045 (6)	−0.0070 (6)	−0.0020 (7)
C12	0.0481 (8)	0.0597 (9)	0.0386 (7)	0.0038 (6)	0.0011 (6)	−0.0071 (6)
C13	0.0353 (6)	0.0406 (7)	0.0383 (6)	0.0003 (5)	0.0087 (5)	0.0010 (5)
C14	0.0585 (9)	0.0879 (13)	0.0420 (8)	−0.0129 (9)	0.0003 (7)	0.0006 (8)
C15	0.0411 (7)	0.0427 (7)	0.0363 (6)	0.0042 (6)	0.0088 (5)	−0.0019 (5)
C16	0.0559 (8)	0.0505 (9)	0.0623 (9)	0.0119 (7)	0.0237 (7)	0.0108 (7)

### Geometric parameters (Å, º)

**Table:**

N1—C15	1.2761 (16)	C8—C9	1.3917 (17)
N1—C13	1.4204 (15)	C8—C13	1.4039 (17)
F1—C10	1.3673 (15)	C9—C10	1.375 (2)
C1—C2	1.382 (2)	C9—H9	0.9300
C1—C6	1.3890 (19)	C10—C11	1.368 (2)
C1—H1	0.9300	C11—C12	1.382 (2)
C2—C3	1.370 (3)	C11—H11	0.9300
C2—H2	0.9300	C12—C13	1.3892 (19)
C3—C4	1.378 (3)	C12—H12	0.9300
C3—H3	0.9300	C14—H14A	0.9600
C4—C5	1.387 (2)	C14—H14B	0.9600
C4—H4	0.9300	C14—H14C	0.9600
C5—C6	1.381 (2)	C15—C16	1.4992 (19)
C5—H5	0.9300	C15—C15^i^	1.502 (2)
C6—C7	1.5231 (18)	C16—H16A	0.9600
C7—C8	1.5200 (18)	C16—H16B	0.9600
C7—C14	1.537 (2)	C16—H16C	0.9600
C7—H7	0.9800		
			
C15—N1—C13	119.34 (11)	C10—C9—H9	120.3
C2—C1—C6	121.28 (17)	C8—C9—H9	120.3
C2—C1—H1	119.4	F1—C10—C11	118.60 (13)
C6—C1—H1	119.4	F1—C10—C9	118.19 (13)
C3—C2—C1	120.33 (16)	C11—C10—C9	123.20 (12)
C3—C2—H2	119.8	C10—C11—C12	117.84 (13)
C1—C2—H2	119.8	C10—C11—H11	121.1
C2—C3—C4	119.17 (16)	C12—C11—H11	121.1
C2—C3—H3	120.4	C11—C12—C13	120.79 (13)
C4—C3—H3	120.4	C11—C12—H12	119.6
C3—C4—C5	120.61 (17)	C13—C12—H12	119.6
C3—C4—H4	119.7	C12—C13—C8	120.45 (12)
C5—C4—H4	119.7	C12—C13—N1	119.94 (12)
C6—C5—C4	120.71 (15)	C8—C13—N1	119.45 (11)
C6—C5—H5	119.6	C7—C14—H14A	109.5
C4—C5—H5	119.6	C7—C14—H14B	109.5
C5—C6—C1	117.87 (14)	H14A—C14—H14B	109.5
C5—C6—C7	121.83 (12)	C7—C14—H14C	109.5
C1—C6—C7	120.30 (13)	H14A—C14—H14C	109.5
C8—C7—C6	111.74 (11)	H14B—C14—H14C	109.5
C8—C7—C14	113.17 (12)	N1—C15—C16	126.30 (12)
C6—C7—C14	111.80 (12)	N1—C15—C15^i^	116.24 (15)
C8—C7—H7	106.5	C16—C15—C15^i^	117.44 (14)
C6—C7—H7	106.5	C15—C16—H16A	109.5
C14—C7—H7	106.5	C15—C16—H16B	109.5
C9—C8—C13	118.26 (12)	H16A—C16—H16B	109.5
C9—C8—C7	122.99 (12)	C15—C16—H16C	109.5
C13—C8—C7	118.74 (11)	H16A—C16—H16C	109.5
C10—C9—C8	119.41 (12)	H16B—C16—H16C	109.5
			
C6—C1—C2—C3	0.2 (3)	C7—C8—C9—C10	−178.02 (13)
C1—C2—C3—C4	1.3 (3)	C8—C9—C10—F1	178.66 (12)
C2—C3—C4—C5	−1.6 (3)	C8—C9—C10—C11	−0.3 (2)
C3—C4—C5—C6	0.4 (3)	F1—C10—C11—C12	−179.60 (13)
C4—C5—C6—C1	1.1 (2)	C9—C10—C11—C12	−0.7 (2)
C4—C5—C6—C7	−178.53 (14)	C10—C11—C12—C13	0.0 (2)
C2—C1—C6—C5	−1.4 (2)	C11—C12—C13—C8	1.7 (2)
C2—C1—C6—C7	178.26 (16)	C11—C12—C13—N1	177.16 (13)
C5—C6—C7—C8	51.52 (17)	C9—C8—C13—C12	−2.62 (19)
C1—C6—C7—C8	−128.13 (14)	C7—C8—C13—C12	177.29 (12)
C5—C6—C7—C14	−76.48 (18)	C9—C8—C13—N1	−178.09 (11)
C1—C6—C7—C14	103.86 (17)	C7—C8—C13—N1	1.83 (18)
C6—C7—C8—C9	−107.17 (14)	C15—N1—C13—C12	77.96 (17)
C14—C7—C8—C9	20.09 (19)	C15—N1—C13—C8	−106.56 (14)
C6—C7—C8—C13	72.91 (15)	C13—N1—C15—C16	2.1 (2)
C14—C7—C8—C13	−159.82 (13)	C13—N1—C15—C15^i^	−179.36 (13)
C13—C8—C9—C10	1.89 (19)		

Symmetry code: (i) −*x*+1, −*y*+1, −*z*+1.
